# Selective chemical binding enhances cesium tolerance in plants through inhibition of cesium uptake

**DOI:** 10.1038/srep08842

**Published:** 2015-03-05

**Authors:** Eri Adams, Vitaly Chaban, Himanshu Khandelia, Ryoung Shin

**Affiliations:** 1 RIKEN Center for Sustainable Resource Science, 1-7-22 Suehirocho, Tsurumi-ku, Yokohama, Kanagawa 230-0045, Japan; 2 MEMPHYS, Center for BioMembrane Physics, University of Southern Denmark, Campusvej 55, Odense M 5230, Denmark

## Abstract

High concentrations of cesium (Cs^+^) inhibit plant growth but the detailed mechanisms of Cs^+^ uptake, transport and response in plants are not well known. In order to identify small molecules with a capacity to enhance plant tolerance to Cs^+^, chemical library screening was performed using *Arabidopsis*. Of 10,000 chemicals tested, five compounds were confirmed as Cs^+^ tolerance enhancers. Further investigation and quantum mechanical modelling revealed that one of these compounds reduced Cs^+^ concentrations in plants and that the imidazole moiety of this compound bound specifically to Cs^+^. Analysis of the analogous compounds indicated that the structure of the identified compound is important for the effect to be conferred. Taken together, Cs^+^ tolerance enhancer isolated here renders plants tolerant to Cs^+^ by inhibiting Cs^+^ entry into roots via specific binding to the ion thus, for instance, providing a basis for phytostabilisation of radiocesium-contaminated farmland.

Cesium (Cs^+^) exists naturally at relatively low levels of approximately 3 ppm^1^ in the Earth's crust but it is not required by plants. Because of this, Cs^+^ has long been overlooked in the field of plant biology other than for its function as a potassium (K^+^) channel blocker^2^. However, the recent accident at the Fukushima Daiichi Nuclear Power Plant in Japan following the great earthquake and tsunami resulted in widespread radioactive contamination of farmland and created a pressing need for greater understanding of the mechanisms by which plants absorb and transport Cs^+^ from soil. Cs-137 and Cs-134 with a relatively long half-life of 30.17 and 2.07 years, respectively, pose the major health threat among the various isotopes of radiocesium distributed after nuclear accidents. The high solubility of Cs^+^ in water increases the risk of Cs^+^ entering the food chain especially through agricultural crops grown in contaminated soils. In plants, Cs^+^ has no known beneficial function but high concentrations of Cs^+^ can cause major retardation of plant growth. This, in turn, suggests that plants have the ability to absorb and accumulate high levels of Cs^+^, high enough to disturb normal growth, although it is unlikely that they have a specific system devoted to transport Cs^+^. The fact that plants can readily absorb Cs^+^ is a threat from an agricultural point of view, and to ensure the safety of agricultural products grown in the contaminated soils, molecular understanding of Cs^+^ uptake, accumulation and responses in plants is essential but current knowledge is limited. It is considered that monocation uptake systems, especially K^+^, which belongs to the same alkali I metal group as Cs^+^, are the major point of entry for Cs^+^ at the root-soil interface such as voltage-insensitive cation channels (VICCs) and K^+^ uptake permeases (KUPs)^3^. In a model plant, *Arabidopsis thaliana*, members of the KUP family, HIGH AFFINITY K^+^ TRANSPORTER5 (HAK5) and KUP9 have been indicated to be involved in Cs^+^ uptake^4^,^5^ and a change in amino acids of CNGC1 has been associated with high Cs^+^ accumulation^6^. The fine balance of K^+^ and Cs^+^ concentrations are known to affect plant performance since Cs^+^ disturbs various physiological processes by competing with K^+^ in the cell^7^,^8^. Growth retardation caused by high concentrations of Cs^+^ has been shown to be, at least partly, mediated through the jasmonate pathway, a phytohormone pathway which is mainly involved in biotic and abiotic stress responses^9^. Recent studies have also suggested that Cs^+^ is sequestrated in vacuoles and a SNARE protein SEC22 may be responsible for Cs^+^ deposition in vacuoles^10^,^11^.

In practice, various factors are suggested to alter the degree of Cs^+^ accumulation in plants such as plant species/cultivars, Ca^2+^ levels, plant-associated microorganisms and Cs^+^ location in the soil as well as atmospheric CO_2_ levels^12^,^13^,^14^,^15^,^16^. In order to alter Cs^+^ uptake, fundamental modification of the plant system such as genetic modification or chemical treatment is considered to be essential. To elucidate the fundamentals involved, a chemical library screening was performed to select the chemicals which could render plants tolerant to Cs^+^. Of the selected chemicals, those which confer tolerance due to lower accumulation of Cs^+^ in *Arabidopsis*, can also be expected to lower radiocesium accumulation in agricultural crops. The advantage of chemical application to contaminated soils is that the effect is most likely universal for all plant species unlike a genetic modification which might only work for certain taxa. The high-throughput chemical screenings that have been undertaken successfully isolated some chemicals useful for application in the field such as a series of immune-priming compounds for crop protection^17^,^18^,^19^. In this study, we isolated five chemical compounds from the commercial Maybridge chemical collection comprised of 10,000 small organic compounds which act as Cs^+^ tolerance enhancers in *Arabidopsis*, herein referred to as CsTolens. Of the five, one chemical, CsTolen A, was shown to reduce Cs^+^ accumulation in plants. Further investigation and theoretical modelling suggested that CsTolen A binds to Cs^+^, inhibiting its entry and thereby provides the plant with tolerance.

## Results

### Screening for chemicals rendering plants tolerant to Cs^+^

**Figure 1:**
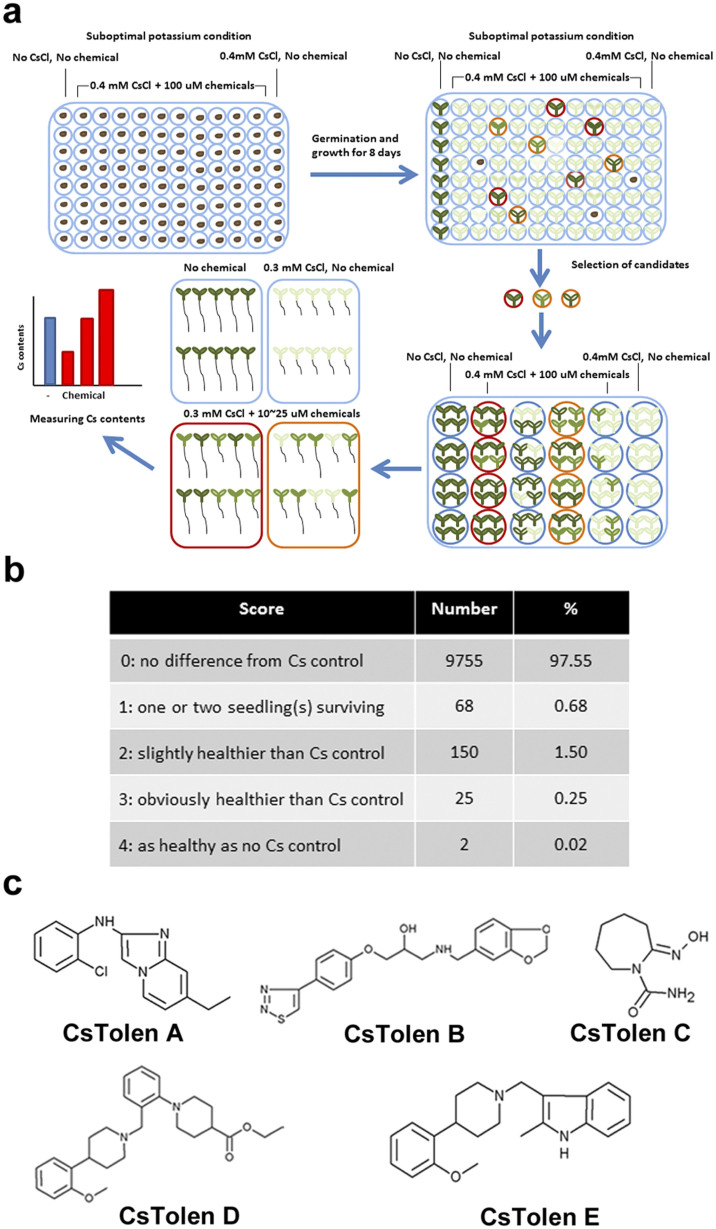
Chemical library screening. (a) Schematic representation of chemical screening. Wild type (Col-0) seeds were sown on 0.5 mM KCl + 0.4 mM CsCl media containing 100 μM chemical and grown for 8 days prior to scoring. Those chemicals which rendered plants tolerant to Cs^+^ were chosen as candidates. Further screenings were performed on a larger scale. Cs^+^ concentrations in the seedlings were determined at the final screening. (b) Scoring results of the initial screening. Number and the percentage of chemicals which fall into each score are shown. (c) Chemical structures of Cs^+^ tolerance enhancers (CsTolen A–E). The candidate chemicals with score >1 were selected and analysed through four steps of screenings. Consequently, five chemicals were confirmed to enhance plant tolerance to Cs^+^.

High concentrations of Cs^+^ can inhibit plant growth and cause chlorosis of the aerial parts. In order to isolate the chemicals which alter plant responses to Cs^+^, Maybridge (http://www.maybridge.com/) chemical library comprising 10,000 small synthetic compounds was screened for enhanced tolerance of *Arabidopsis thaliana* seedlings to Cs^+^. The screening was performed in a stringent condition, 0.5 mM KCl + 0.4 mM CsCl, where most of the control plants could not survive. Each chemical was assessed for its ability to reverse the deleterious effects of Cs^+^ when added to the growth media (final concentration of 100 μM). A suboptimal K^+^ condition (0.5 mM) was used instead of an optimal condition (1.75 mM) since the effects of Cs^+^ were more distinct at the higher Cs^+^/K^+^ ratios. A scoring system was established to "quantify" the visual phenotype compared to that of the control plants: score zero; no difference from Cs^+^-treated control, score one; one or two seedling(s) surviving, score two; slightly healthier than Cs^+^ control, score three; obviously healthier than Cs^+^ control and score four; as healthy as no Cs^+^ control. The plate layout and the general scheme of the screening are shown in Fig. 1a. According to the scoring system, the initial screening identified 245 chemicals with score equal to or more than one (Fig. 1b). The second, third and fourth screenings narrowed the number down to five chemicals which were confirmed to render plants tolerant to high concentrations of Cs^+^ (Fig. 1c).

### Effects of the selected Cs^+^ tolerance enhancers in plants

**Figure 2:**
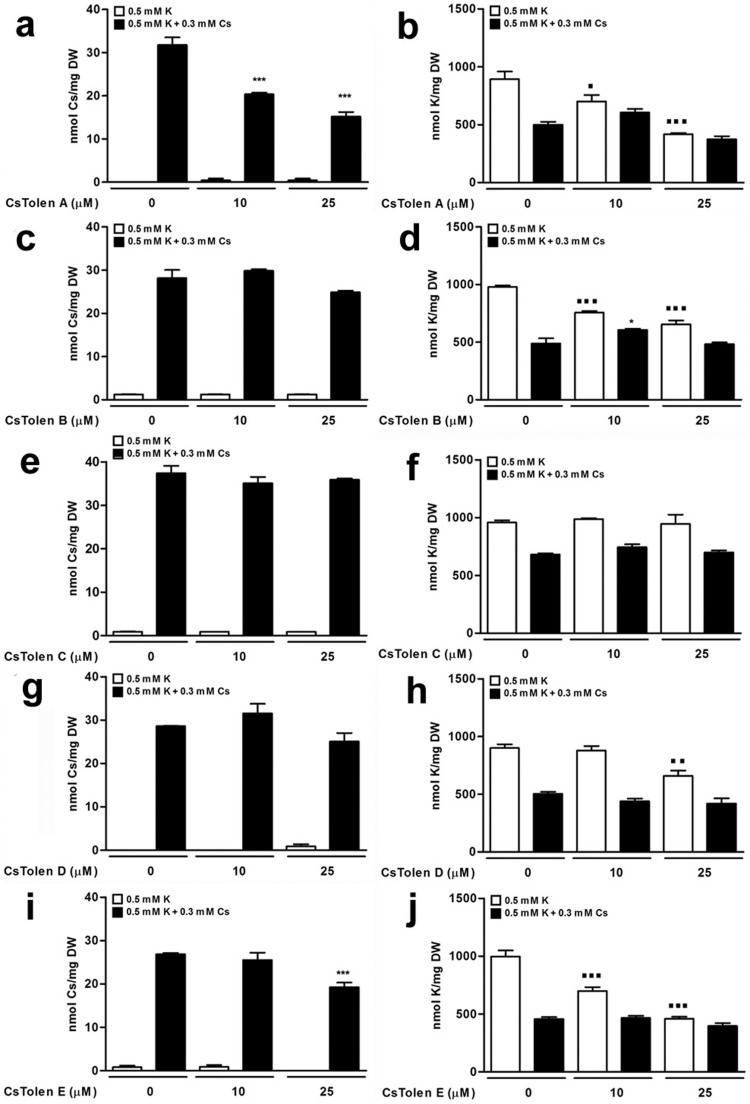
Cs^+^ and K^+^ concentrations in Col-0 treated with Cs^+^ and CsTolens. (a) Cs^+^ and (b) K^+^ concentrations in plants treated with CsTolen A. (c) Cs^+^ and (d) K^+^ concentrations in plants treated with CsTolen B. (e) Cs^+^ and (f) K^+^ concentrations in plants treated with CsTolen C. (g) Cs^+^ and (h) K^+^ concentrations in plants treated with CsTolen D. (i) Cs^+^ and (j) K^+^ concentrations in plants treated with CsTolen E. Plants grown on suboptimal (0.5 mM) KCl media in the presence or absence of 0.3 mM CsCl and 10 or 25 μM of CsTolens for 8 days were dried and extracted. The symbols indicate statistically significant differences compared to non-CsTolen controls: black squares for 0.5 mM KCl and asterisks for 0.5 mM KCl + 0.3 mM CsCl. The number of symbols donates the significance of the difference (n = 3): three for *P* < 0.001, two for *P* < 0.01 and one for *P* < 0.05.

**Figure 3:**
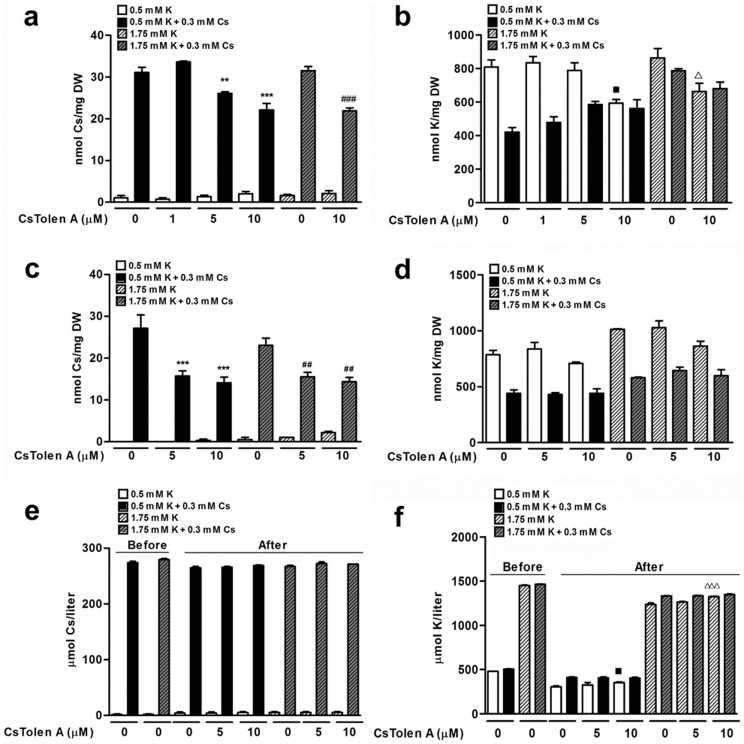
Cs^+^ and K^+^ concentrations in Col-0 treated with CsTolen A. (a) Cs^+^ and (b) K^+^ concentrations in plants grown on solid media. Plants germinated and grown on suboptimal (0.5 mM) and optimal (1.75 mM) KCl media in the presence or absence of 0.3 mM CsCl and the indicated concentrations of CsTolen A for 8 days were dried and extracted. (c) Cs^+^ and (d) K^+^ concentrations in plants grown in liquid media. Plants germinated and grown in liquid suboptimal (0.5 mM) and optimal (1.75 mM) KCl media in the presence or absence of 0.3 mM CsCl and the indicated concentrations of CsTolen A for 7 days were dried and extracted. (e) Cs^+^ and (f) K^+^ concentrations in liquid media before and after the experiment. The symbols indicate statistically significant differences compared to non-CsTolen A controls: black squares for 0.5 mM KCl, asterisks for 0.5 mM KCl + 0.3 mM CsCl, white triangles for 1.75 mM KCl and number signs for 1.75 mM KCl + 0.3 mM CsCl. The number of symbols donates the significance of the difference (n = 3): three for *P* < 0.001, two for *P* < 0.01 and one for *P* < 0.05.

The five chemicals which were isolated as enhancing plant tolerance to Cs^+^ through the chemical screening were named CsTolen A–E (Fig. 1c) and each was investigated further for their effects on Cs^+^-treated plants. In the milder condition, 0.5 mM KCl + 0.3 mM CsCl, all CsTolens presented strong recovery from the negative effects of Cs^+^ and CsTolen A–C conferred good recovery even in the most stringent condition (Table 1) although CsTolen C inhibited root growth (Supplementary Fig. 1). Upon elemental analysis, CsTolen A and E were associated with lower accumulation of Cs^+^ in *Arabidopsis* (Fig. 2). All the chemicals except CsTolen C were also associated with lower accumulation of K^+^ in plants (Fig. 2). Further investigation through elemental analysis revealed that CsTolen A reduced accumulation of Cs^+^ in plants at as low as 5 μM (Fig. 3a, photos in Supplementary Fig. 2) and on another front, K^+^ accumulation was reduced at 10 μM (Fig. 3b). This pattern was also observed for plants grown in a liquid culture except that the reduction of K^+^ concentrations was not sufficient to give statistical difference (Fig. 3c,d). Analysis of the liquid growth media before and after the experiment revealed that Cs^+^ concentrations were significantly reduced after the experiment having been absorbed by the plants (*P* < 0.05 at suboptimal K^+^ and *P* < 0.01 at optimal K^+^) but a reduction of Cs^+^ in the media was not statistically significant when CsTolen A was added, confirming lower Cs^+^ uptake by the plants in the presence of CsTolen A (Fig. 3e). By contrast, a reduction of K^+^ concentrations in the growth media after the experiment were statistically significant for all the treatments relative to ones before, both in the presence or absence of Cs^+^ (*P* < 0.001), but more K^+^ remained in the media when 10 μM CsTolen A was added in the absence of Cs^+^ (*P* < 0.05 at suboptimal K^+^ and *P* < 0.001 at optimal K^+^; Fig. 3f).

### Quantum mechanical modelling of CsTolen A with Cs^+^ and other alkali metals

The mechanism for the reduced accumulation of Cs^+^ in plants conferred by CsTolen A is only speculative but it may be that CsTolen A inhibits Cs^+^ entry into the roots or promotes Cs^+^ extrusion from plant cells. To clarify whether the field of action of CsTolen A is external or internal, a transfer assay was performed. Plants germinated in the presence or the absence of 10 μM CsTolen A were transferred onto media containing 0.3 mM CsCl. Pre-treatment with CsTolen A did not change the concentrations of Cs^+^ or K^+^ in the plants (Supplementary Fig. 3), suggesting that CsTolen A contributes to plant tolerance through inhibition of Cs^+^ influx into the plants by either blocking transporters/channels for Cs^+^ or binding Cs^+^ to derange its capability for passing through the transporters/channels.

**Figure 4:**
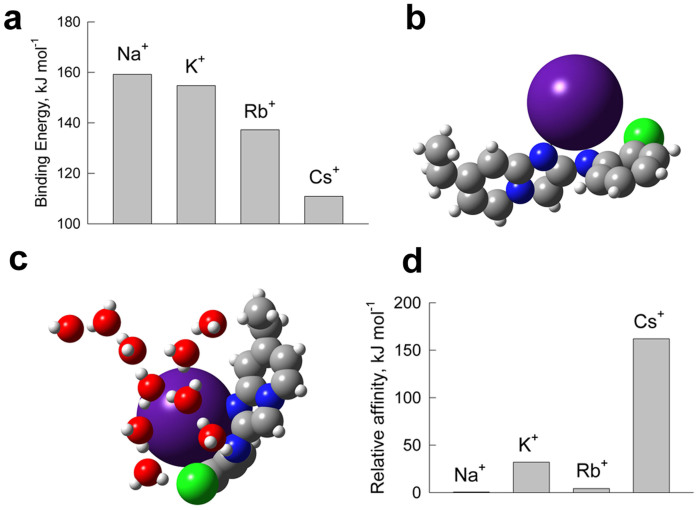
Quantum mechanical modelling of binding between CsTolen A and alkali metals. (a) Binding energy of CsTolen A with each alkali metal in the aqueous system. (b) Schematic representation of the suggested binding model between CsTolen A and Cs^+^. (c) Schematic representation of the suggested binding model between CsTolen A and Cs^+^ in the presence of water molecules. (d) Relative affinity of CsTolen A to each alkali metal in the presence of water molecules. The difference of the binding energies of each alkali metal to CsTolen A compared to the first hydration shell is plotted relative to Na^+^.

In order to assess the binding capacity between CsTolen A and Cs^+^, quantum mechanical modelling was performed. All the alkali metal ions investigated (K^+^, Na^+^, Rb^+^ and Cs^+^) were shown through hybrid density functional theory numerical modelling to bind strongly to CsTolen A in an aqueous system (Fig. 4a). The basis set superposition error (BSSE)-corrected binding energies were of the same order of magnitude as the electrostatic binding for ion pairs in monovalent salts such as NaCl and KCl and were approximately half of those of ordinary covalent bonds. As expected, the binding strength decreased as ionic diameter increased. All ions were found to be coordinated by the nitrogen atom of imidazole moiety which possesses an excess electrostatic charge of −0.25e, based on Hirshfeld charge assignment, with a weak contribution from the nitrogen of NH in CsTolen A. The binding can therefore be explained by electrostatic interaction of the imidazole part (−0.25e) and the alkali metal (+1e) (Fig. 4b). The chlorine atom does not participate in cation coordination. This is partially due to a small partial charge on chlorine, −0.10e, in this compound. The binding of the imidazole moiety of CsTolen A and all investigated ions in water is somewhat weaker than in a vacuum (Supplementary Fig. 4) due to competitive coordination between water and the chemical. The formation energy of Cs^+^ and CsTolen A structure amounts to 235 kJ mol^−1^, which is comparable to the attraction energy of Cs^+^ to its first hydration shell (304 kJ mol^−1^). A significant amount of valence electron transfer from Cs^+^ to the chemical (−0.46e) was also recorded, which would further stabilise the binding. It should be noted that partial coordination of Cs^+^ by water molecules would be expected in real systems since water molecules would be able to occupy voids due to both thermal motion and enthalpy gain (Fig. 4c). Although smaller alkali cations (Na^+^, K^+^ and Rb^+^) exhibited stronger binding capacity to CsTolen A, selectivity towards Cs^+^ was observed as a result of its relatively weak hydration degree (Fig. 4d). The first hydration shell was selected as a model for the hydrated ions, as particularly these interactions reflect the difference between various ions (Na^+^, K^+^, Rb^+^, Cs^+^). Note, that total binding energy is significantly different in the case of the first hydration shell and in the case of infinitely dilute solution of the ion. Therefore, the numbers in Fig. 4 must not be confused with the ion hydration free energies available elsewhere. The energetic difference between binding of Cs^+^ to CsTolen A and to the first hydration shell is 162 kJ mol^−1^ and 130 kJ mol^−1^ larger than those of Na^+^ and of K^+^, respectively, suggesting that CsTolen A preferentially binds to Cs^+^ in an aqueous solution.

### Effect of structural analogues of CsTolen A on Cs^+^ accumulation

**Figure 5:**
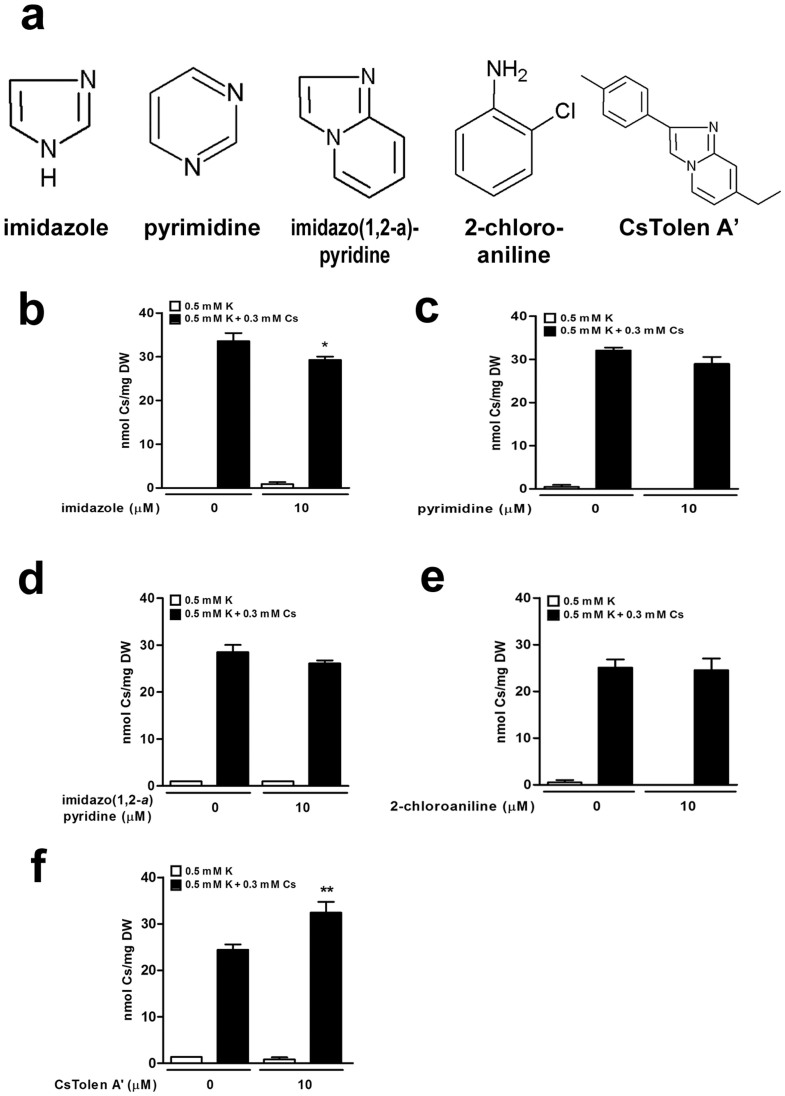
Effects of analogous chemicals of CsTolen A to Cs^+^ accumulation in plants. (a) Chemical structures of the analogous chemicals of CsTolen A tested. (b) Cs^+^ concentrations of Col-0 treated with imidazole, (c) pyrimidine, (d) imidazo(1,2-*a*)pyridine, (e) 2-chloroaniline and (f) CsTolen A'. Plants germinated and grown on suboptimal (0.5 mM) KCl media in the presence or absence of 0.3 mM CsCl and 10 μM of chemicals for 8 days were dried and extracted. Asterisks indicate statistically significant differences compared to the non-CsTolen control: 0.5 mM KCl + 0.3 mM CsCl. The number of symbols donates the significance of the difference (n = 3): two for *P* < 0.01 and one for *P* < 0.05.

Theoretical modelling predicted that the imidazole moiety of CsTolen A would be the site of Cs^+^ binding. However, exogenous application of imidazole or pyrimidine did not reverse the growth inhibition caused by Cs^+^ and Cs^+^ accumulation was only slightly reduced by imidazole (*P* < 0.05) although imidazole, pyrimidine or pyridine have ability to bind to Cs^+^ according to the theoretical modelling (Fig. 5a,b,c; Supplementary Fig. 5). By contrast, imidazo(1,2-*a*)pyridine contributed to a mild recovery from the Cs^+^ damage but accumulation of Cs^+^ was not reduced (Fig. 5a,d; Supplementary Fig. 5). The counterpart of the identified chemical, 2-chloroaniline was shown to have no effect on Cs^+^-treated plants (Fig. 5a,e; Supplementary Fig. 5). These results indicated that binding of Cs^+^ to the imidazole ring alone might not be sufficient to render plants tolerant to Cs^+^. A structural analogue CsTolen A' which has an imidazo(1,2-*a*)pyridine backbone and a similar size to CsTolen A was analysed, however neither did the Cs^+^ concentrations in plants decline (rather they increased a little, *P* < 0.01) nor did the plants gain any tolerance (Fig. 5a,f; Supplementary Fig. 5). This observation corresponded with the theoretical modelling which indicated approximately 30 kJ mol^−1^ less binding affinity between CsTolen A' and Cs^+^ compared to the one between CsTolen A and Cs^+^ (Fig. 4d; Supplementary Fig. 5) probably because of the second nitrogen atom in CsTolen A that contributed to binding.

### Specific effects of CsTolen A to Cs^+^ stress

According to our theoretical modelling, CsTolen A binds strongly to other alkali metal ions such as K^+^ and Na^+^. Salt stress caused by Na^+^ is a major concern in agriculture. However, CsTolen A did not reverse Na^+^-induced growth retardation as it did for Cs^+^ (Supplementary Fig. 6). Similarly, CsTolen A did not reverse K^+^ deficiency phenotype, which is another concern in agriculture (Supplementary Fig. 6). CsTolen A also did not reverse growth inhibition phenotype caused by high concentrations of K^+^ or Rb^+^ (Supplementary Fig. 6). Elemental analysis of Na^+^-treated plants indicated that a small reduction of Na^+^ concentrations (*P* < 0.01 at optimal K^+^ and not statistically significant at suboptimal K^+^) was observed (Supplementary Fig. 7). Na^+^ caused a significant reduction of K^+^ concentrations as expected but addition of CsTolen A did not further alter K^+^ concentrations (Supplementary Fig. 7).

### Application of CsTolen A to soil-grown plants

**Figure 6:**
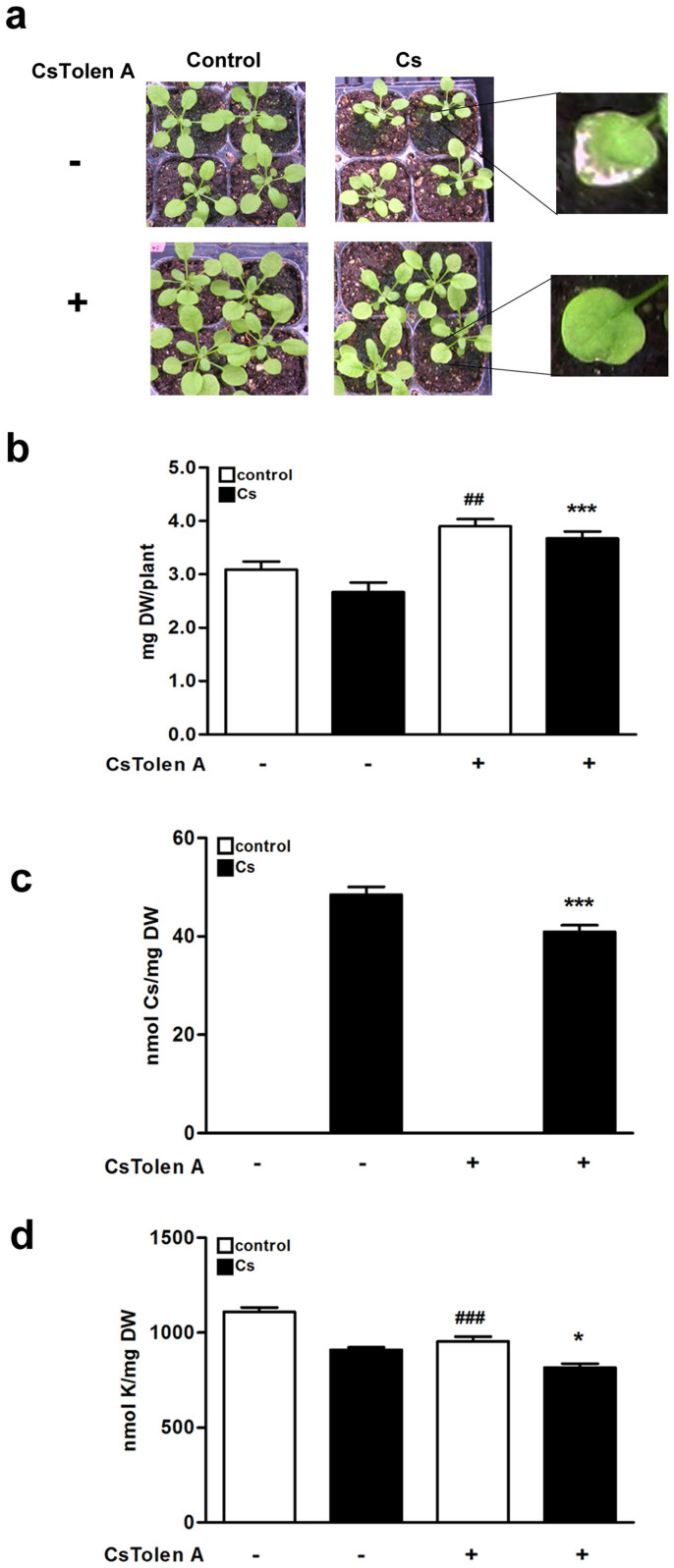
Soil-grown Col-0 treated with CsTolen A. (a) Images, (b) Dry weight, (c) Cs^+^ and (d) K^+^ concentrations of soil-grown 21-day-old Col-0 plants treated with Cs^+^ and CsTolen A. Plants were germinated and grown on soil mixed with 0 or 10.8 mmol CsCl kg^−1^ dry soil and treated with CsTolen A (1.25 μmol kg^−1^ soil each time) on Day 0, 7 and 14. Aerial parts were harvested on Day 21, dried and extracted. The symbols indicate statistically significant differences compared to non-CsTolen A controls: number signs for non-Cs^+^ control and asterisks for Cs^+^-treated control. The number of symbols donates the significance of the difference (n = 16): three for *P* < 0.001, two for *P* < 0.01 and one for *P* < 0.05.

Further analysis was performed to demonstrate the effectiveness of CsTolen A for plants germinated and grown in soil. *Arabidopsis* seeds were directly germinated in soil containing Cs^+^ (approximately 10.8 mmol Cs^+^ kg^−1^ dry soil) and CsTolen A treatment was applied once weekly for three weeks. CsTolen A treatment improved plant performance in the presence or absence of Cs^+^ such as increased dry weight and eliminated chlorosis on the rosette leaves caused by high concentrations of Cs^+^ (Fig. 6a,b). Cs^+^ concentrations were significantly reduced in plants treated with CsTolen A, confirming the effectiveness of the chemical in soil-grown plants (Fig. 6c). K^+^ concentrations were only mildly reduced (Fig. 6d). This trend was also observed in different developmental stages, different concentrations of Cs^+^ (7.2 and 12.96 mmol Cs^+^ kg^−1^ dry soil) and different application methods of CsTolen A (photos of the 12.96 mmol Cs^+^ experiment in Supplementary Fig. 8).

## Discussion

The ability of plants to accumulate radionuclides and the use of plants to monitor radiation have long been recognised^20^ but the details of the mechanisms by which plants accumulate Cs^+^ through either: foliar absorption or root uptake is still obscure. Following the immediate radiation fallout deposition from a nuclear accident, root uptake becomes more of a long-term concern. Consequently, an understanding of how plants absorb and respond to Cs^+^ can help identify ways to eliminate harmful radioactive substances from food chain and also remediate the contaminated area using plants. As a result of the chemical library screening performed, we have successfully isolated five chemicals out of 10,000 that enhance plant tolerance to high concentrations of Cs^+^, herein called CsTolen A–E. These are small organic compounds with a molecular weight of around 200–400. No particular structural similarities among the compounds was observed except that they all contain multiple nitrogen atoms. CsTolen A–C conferred good plant recovery even at the stringent Cs^+^ condition although CsTolen C inhibited root growth. Enhanced tolerance conferred by CsTolen B–D was not due to reduced Cs^+^ accumulation in the plants therefore, these chemicals could be involved in the Cs^+^ perception and response pathways in plants. Meanwhile, CsTolen A and E which inhibited Cs^+^ accumulation in plants could be beneficial to reduce radiocesium levels in agricultural crops.

Above all, CsTolen A exhibited the strongest antagonistic effects to growth retardation caused by Cs^+^. CsTolen A reduced both Cs^+^ and K^+^ concentrations in *Arabidopsis* but the reduction was most effective for Cs^+^ and this, most likely, contributed to improved tolerance. Transfer experiment and theoretical modelling revealed that CsTolen A binds to Cs^+^ and inhibits the entry of Cs^+^ into the plant. In concert with the theoretical modelling, CsTolen A did not enhance tolerance to Na^+^, Rb^+^, K^+^ stress or K^+^ deficiency and contributed to only a minor reduction of Na^+^ and K^+^ concentrations in the plants. Instead, CsTolen A preferentially bound to Cs^+^ among alkali metals in an aqueous system and enhanced tolerance in plants. Theoretical modelling and analysis of the analogues also suggested that although nitrogen atoms and imidazo(1,2-*a*)pyridine structure were important for Cs^+^ binding, binding alone did not account for the lower Cs^+^ accumulation and enhanced tolerance in plants. This could be due to the specific chemical properties of CsTolen A such as structure, size and charge being particularly suitable for blocking Cs^+^ uptake machineries in plants. Organic synthesis of modified CsTolen A with the aid of theoretical calculations might produce even more effective tolerance enhancers in the future. The advantage of chemical treatment involves a high degree of freedom of choice and the potential of improvement especially where the mode of action of an effective compound is known. Further investigation of the detailed site of action of the related compounds might reveal the Cs^+^ entry points in the roots, very likely proteins, which should be responsible for Cs^+^ uptake in plants.

Applicability of CsTolen A to a soil setting, rather than a laboratory setting, is extremely encouraging. Previous reports have indicated that K^+^ and Ca^2+^ supplementation in soil and arbuscular mycorrhizal (AM) fungus infection in *Medicago truncatula* reduce Cs^+^ accumulation in plants^13^,^14^. However, the effects of Ca^2+^ supplementation are variable among plant species and tissues and are only confirmed in the acid soil^13^. Ability of AM fungi on radiocesium is also heavily dependent on various factors such as host plant species, soil type and nutrient status and conflicting results have been reported^14^. Above all, these treatments are unlikely to function selectively to Cs^+^. By contrast, it is a general agreement that effects of the chemical treatment are universal across most of the plant species, especially when the chemical works outside of the plants as is the case of CsTolen A. Although various compounds and materials such as zeolites, silicates and crown ethers have been proposed as selective Cs^+^-capturing agents for the decontamination of radioactive waste water^21^,^22^,^23^,^24^,^25^,^26^,^27^,^28^, this is the first report, to the best of our knowledge, on a compound which, through selective binding, enables a plant to avoid Cs^+^ uptake and thus harmful accumulation. The efficacy of CsTolen A needs to be assessed in the field at Fukushima, the site of the nuclear accident^29^. Since the Cs^+^ concentrations used to test CsTolen A is significantly higher than those in the contaminated area, its function at the lower Cs^+^ concentrations must be confirmed in the future. Toxicity of CsTolen A also needs to be carefully investigated so that it does not exert a harmful impact on the environment and the human body. The practical application of CsTolen A to ensure safety of agricultural crops harvested from soils with low levels of radiocesium and work to expand our understanding of Cs^+^ uptake mechanisms in plants are tasks for the future.

## Methods

### Plant material and growth conditions

The *Arabidopsis thaliana* (L.) Heynh. accession wild type Col-0 was used. Seeds were surface-sterilised with 70% (v/v) ethanol and 0.05% (v/v) Triton X-100 and sown on media containing optimal (1.75 mM) or suboptimal (0.5 mM) KCl, 2 mM Ca(NO_3_)_2_, 0.5 mM phosphoric acid, 0.75 mM MgSO_4_, 50 μM H_3_BO_3_, 10 μM MnCl_2_, 2 μM ZnSO_4_, 1.5 μM CuSO_4_, 0.075 μM NH_4_Mo_7_O_24_ and 74 μM Fe-EDTA, pH 5.8, with Ca(OH)_2_, 1% (w/v) sucrose and 0.6% (w/v) when the screening was performed and 1% (w/v) for the rest of the experiments of SeaKem agarose (Cambrex) supplemented with designated concentrations of CsCl and other chemicals. After stratification for 3 to 4 days at 4°C, plants were placed in a growth cabinet at 22°C in a 16 h light/8 h dark photocycle with a light intensity of 70–90 μmol m^−2^ sec^−1^. Liquid media were prepared without agarose in Erlenmeyer flasks placed on a shaker in a growth chamber set as 22°C, a 16 h light/8 h dark photocycle.

### Chemical library screening

Maybridge (http://www.maybridge.com/) chemical library with 10,000 small synthetic compounds dissolved in dimethylsulfoxide (DMSO) as 10 mM stock solution was used. Four to five Col-0 seeds were sown in each well containing 100 μl of agar-based growth media and 100 μM chemical in a 96-well plate. Each plate hosted Cs^+^ controls (no chemical added) and no Cs^+^ controls (no CsCl added). Seedlings were grown for 8 days prior to scoring and compared to Cs^+^ and no Cs^+^ controls. The selected candidates based on the scoring system (Fig. 1b) were investigated further and narrowed down through the second, third and fourth screenings. The second screening was performed with three replicates for each chemical in the same scale. The third screening was performed using 24-well plates with each well containing 1.5 ml of growth media, 100 μM chemical and 10 seeds, four replicates for each chemical. The fourth screening was performed using petri dishes containing 50 ml of growth media, 10 and 25 μM chemical and Cs^+^ and K^+^ concentrations in plants were measured.

### Elemental analysis

Seedlings were washed in Milli-Q water, dried on a piece of paper towel, placed in a paper envelope and dried in an oven at 65°C for 3–4 days. Approximately 2 mg of dried samples were extracted in 1 mL of 60% (v/v) HNO_3_ at 125°C for 1 hour, followed by 1 ml of 30% (v/v) H_2_O_2_ and diluted with Milli-Q water to get a total volume of 10 ml. For K^+^ and Na^+^ analysis, samples were further diluted 10 or 100 times with 6% (v/v) HNO_3_. For Cs^+^ analysis, 0.1% (w/v) KCl was added to each sample and standard solution to prevent ionisation of Cs^+^, according to the manufacturer's instructions (PerkinElmer). K^+^ and Cs^+^ concentrations were measured on a flame atomic absorption spectrometer AAnalyst 200 (PerkinElmer). Concentrations were calculated against each standard curve, and one-way ANOVA with Bonferroni's multiple comparison posttest was performed using Prism (GraphPad Software) to determine the statistical significance.

### Theoretical modelling

Density functional theory (DFT) electronic-structure calculations were performed for Na^+^, K^+^, Rb^+^ and Cs^+^ with selected chemicals. Hydration of alkali ions was addressed via binding energy of each ion with 10 surrounding water molecules. The chosen number of water molecules roughly corresponds to the full first hydration shell and partially to the second hydration shell in case of smaller ions, which determines the solvation strength for each ion. Well-trained hybrid DFT functionals are generally free of certain systematic defects and give a significant improvement over pure DFT functionals since these functionals include a mixture of Hartree-Fock exchange with DFT exchange-correlation part. In order to reproduce correct electron transfer and obtain non-biased binding energy between each cation and the chemical, B3LYP^30^,^31^ supplemented by the third-generation (D3) explicit correction for dispersion attraction was used for calculation and the Becke-Johnson damping was applied^32^. The initially constructed geometries of all complexes were optimised prior to binding energy computation. Each initial Z-matrix was adjusted step-by-step, until a stationary point on the potential energy surface was found. Energy and displacement gradients between two consequent geometry optimisation steps were used to conclude that a stationary point was located. Separate convergence criteria were imposed for average (over all atomic positions) and maximum acceptable gradients. The wave function convergence criterion was set to 10^−8^ Ha. No density fitting and other performance improvements were used to preserve accuracy of the results. The atom-centred LanL2DZ basis set^33^,^34^ was chosen, because of heavy elements (beyond K in the periodic table) in the systems. All reported binding energies are corrected with respect to BSSE using the counterpoise approach. The computations were performed in Gaussian 09 program (revision D.01, Gaussian, Inc.), employing in parallel 8, 12 and 16 processor cores per job.

### Soil experiment

Soil experiment was performed in a growth chamber set as 22°C, a 16 h light/8 h dark photocycle. Soil was mixed with CsCl solution to obtain approximately 7.2, 10.8 or 12.96 mmol Cs^+^ kg^−1^ dry soil. Seeds were directly sown and germinated in soil. CsTolen A treatment as 10 μM solution which is equivalent to approximately 1.25 μmol kg^−1^ dry soil each time was performed three times on Day 7, 14 and 21 for the 7.2 mmol Cs^+^ experiment and Day 0, 7 and 14 for the 10.8 mmol Cs^+^ experiment and the aerial part of the seedling was harvested a week after the last CsTolen A treatment for elemental analysis. For the 12.96 mmol Cs^+^ experiment, CsTolen A equivalent to 0.92 μmol kg^−1^ dry soil was mixed in soil before seeds were sown and 10 μM CsTolen A solution was sprayed on the seedlings on Day 7. Images were taken on Day 15.

## Supplementary Material

Supplementary Information Supplementary Figure 1-8

## Figures and Tables

**Table 1:** Recovery of Col-0 treated with Cs^+^ by CsTolen A–E. Plants were germinated and grown in stringent (0.5 mM KCl + 0.4 mM CsCl) and less stringent (0.5 mM KCl + 0.3 mM CsCl) conditions in the presence of the indicated concentrations (μM) of CsTolen A–E for 8 days. The Green% was calculated by counting the number of seedlings which had greening of the cotyledons

			CsTolen				
	0.5 K	0.3Cs	0.3Cs + 10A	0.3Cs + 15B	0.3Cs + 50C	0.3Cs + 15D	0.3Cs + 10E
Total#	38	37	39	39	37	36	37
Green#	38	14	36	30	30	31	28
Green%	100.0	37.8	92.3	76.9	81.1	86.1	75.7

			CsTolen				
	0.5 K	0.4Cs	0.4Cs + 10A	0.4Cs + 15B	0.4Cs + 50C	0.4Cs + 15D	0.4Cs + 10E
Total#	41	44	41	48	43	39	39
Green#	41	3	28	17	21	5	5
Green%	100.0	6.8	68.3	35.4	48.8	12.8	12.8
