# Supplementary material for: Selective chemical binding enhances cesium tolerance in plants through inhibition of cesium uptake
Source: Sci Rep. 2015 Mar 5;5:8842. doi: 10.1038/srep08842 (PMC5390090; doi:10.1038/srep08842)
Supplement: Supplementary Information — Supplementary Figure 1-8 [file srep08842-s1.doc]

**Selective chemical binding enhances cesium tolerance in plants through inhibition of cesium uptake**

Eri Adams^1^, Vitaly Chaban^2^, Himanshu Khandelia^2^ & Ryoung Shin^1^

^1^RIKEN Center for Sustainable Resource Science, 1-7-22 Suehirocho, Tsurumi-ku, Yokohama, Kanagawa 230-0045, Japan

^2^MEMPHYS, Center for BioMembrane Physics, University of Southern Denmark, Campusvej 55, Odense M 5230, Denmark

Correspondence should be addressed to R.S. (ryoung.shin@riken.jp).

**Supplementary Information**


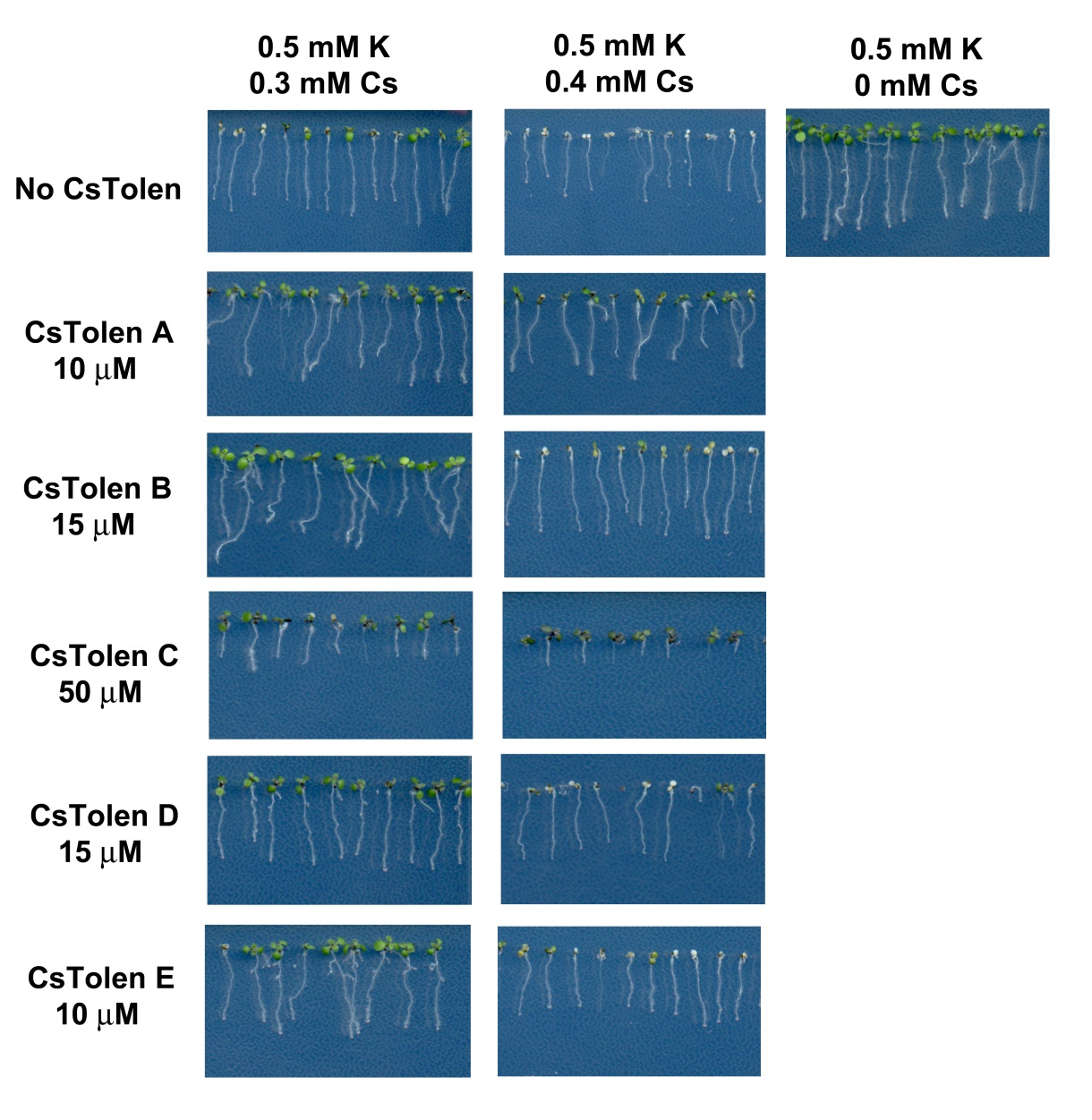


**Supplementary Figure 1. Images of Col-0 treated with Cs^+^ and CsTolens.** Plants were germinated and grown in stringent (0.5 mM KCl + 0.4 mM CsCl) and less stringent (0.5 mM KCl + 0.3 mM CsCl) conditions in the presence of the indicated concentrations of CsTolen A-E for 8 days.


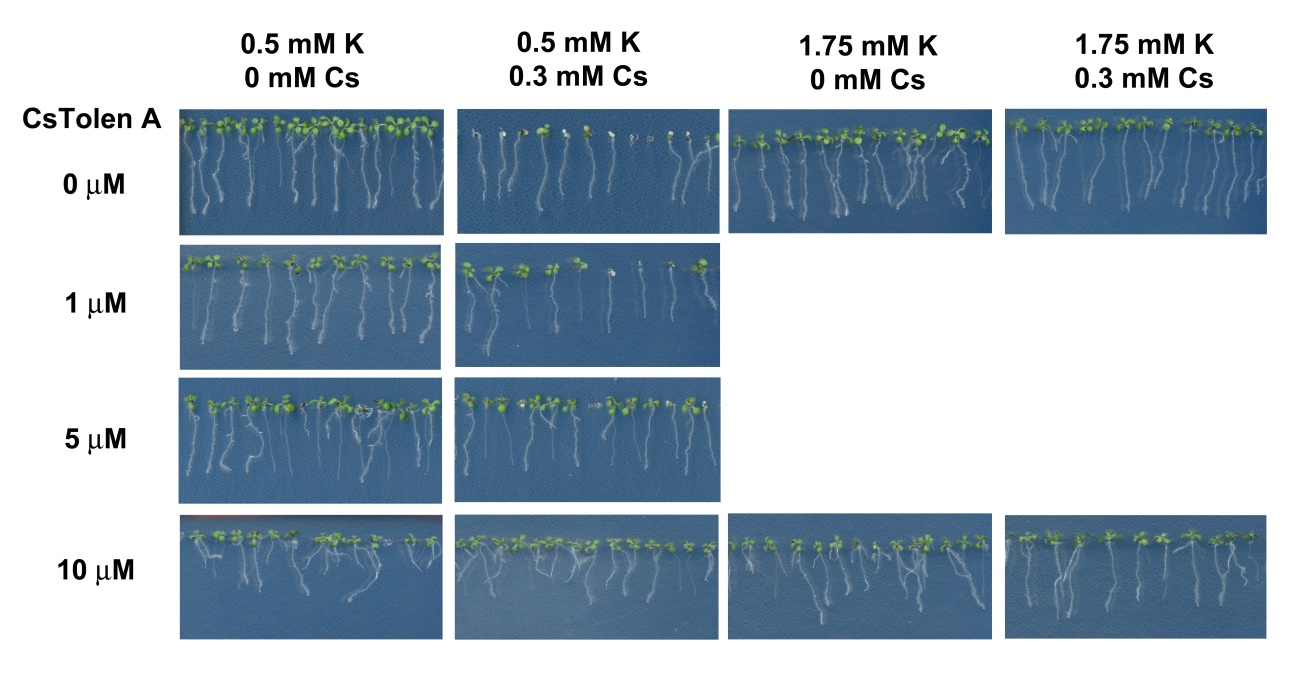


**Supplementary Figure 2. Images of Col-0 treated with Cs^+^ and CsTolen A.** Plants were germinated and grown on suboptimal (0.5 mM) or optimal (1.75 mM) KCl media in the presence or absence of 0.3 mM CsCl and the indicated concentrations of CsTolen A for 8 days.


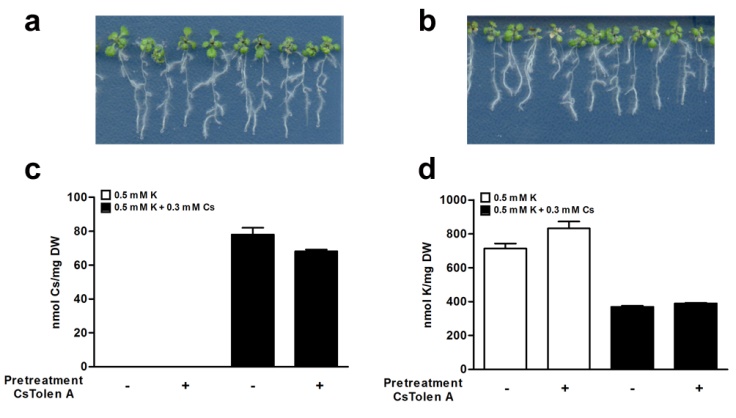


**Supplementary Figure 3. Ability of CsTolen A to function inside plants.** (**a**) Images of Col-0 without or (**b**) with CsTolen A pre-treatment. Plants germinated on suboptimal (0.5 mM) media in the presence or absence of CsTolen A were transferred onto media containing 0.3 mM CsCl on Day 5 and grown for 7 more days prior to imaging. (**c**) Cs^+^ and (**d**) K^+^ concentrations in plants with or without CsTolen A pre-treatment. No statistically significant difference was observed (n > 70 for Cs^+^-treated plants, n > 30 for the control plants, pooled into three technical replicates).


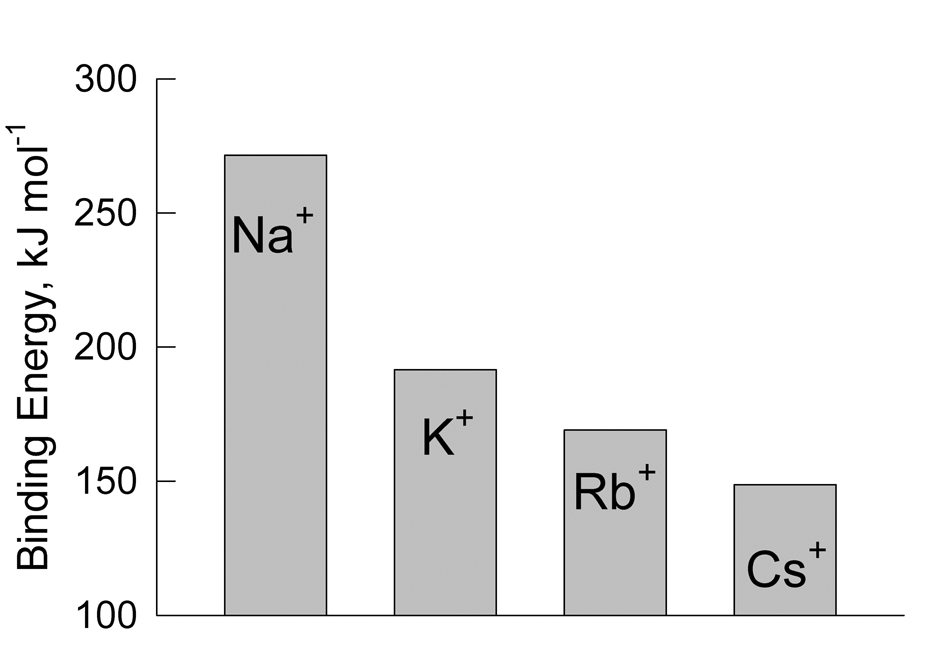


**Supplementary Figure 4. Binding energy of CsTolen A with each alkali metal in a vacuum.**


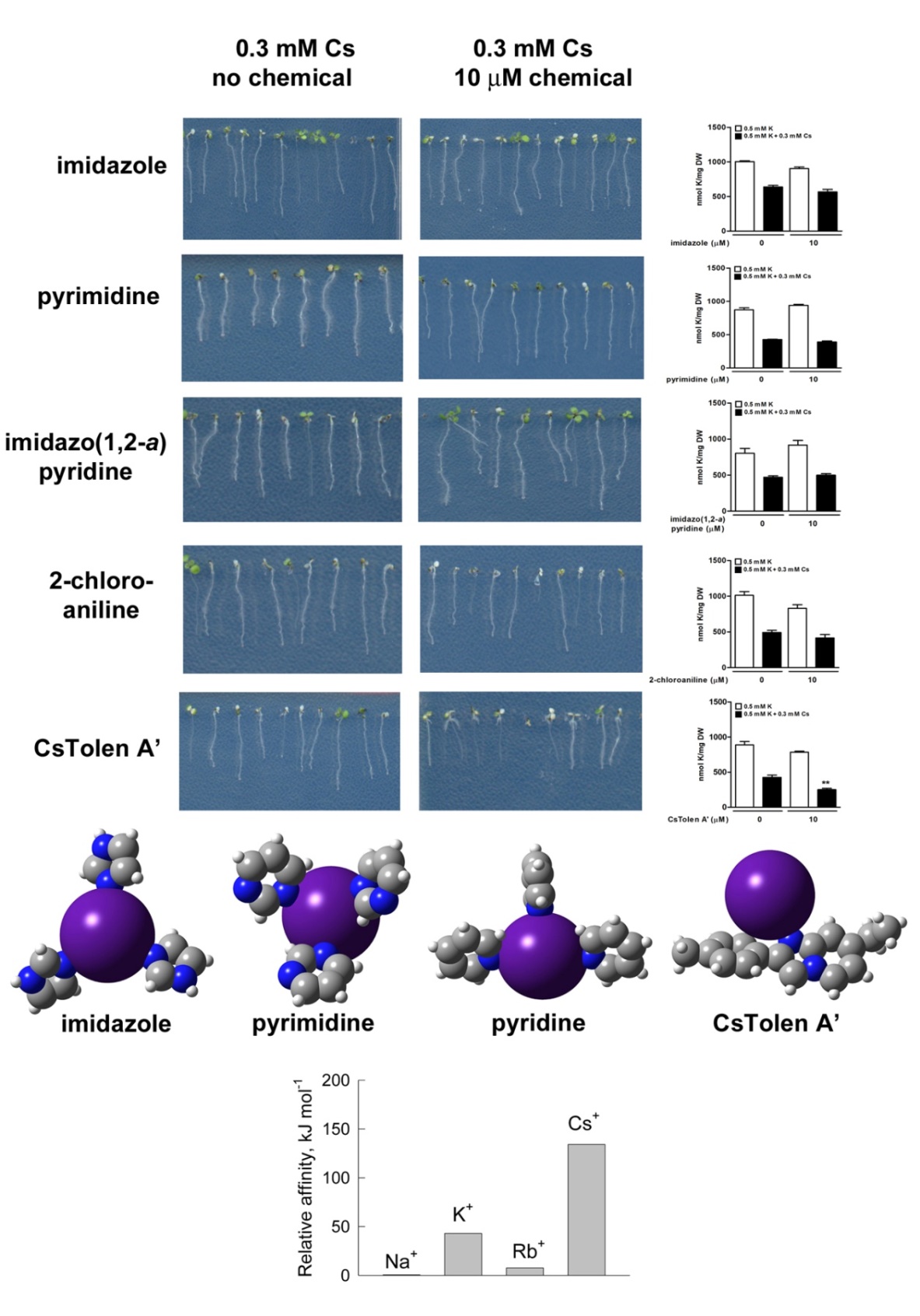


**Supplementary Figure 5. Effect of structural analogues of CsTolen A to Cs^+^-treated Col-0.** Plants grown on suboptimal (0.5 mM) KCl media in the presence or absence of 0.3 mM CsCl and 10 μM chemical for 8 days were dried and extracted. K^+^ concentrations in plants treated with each chemical are shown. The astrisks indicate statistically significant differences compared to non-CsTolen controls: 0.5 mM KCl + 0.3 mM CsCl (n=3, *P* < 0.01). A schematic representation of the suggested binding model between Cs^+^ and imidazole, pyrimidine, pyridine and CsTolen A' is shown. The relative affinity of CsTolen A' to each alkali metal in the presence of water molecules is also shown. The difference of the binding energies of each alkali metal to CsTolen A' compared to the first hydration shell is plotted relative to Na^+^.


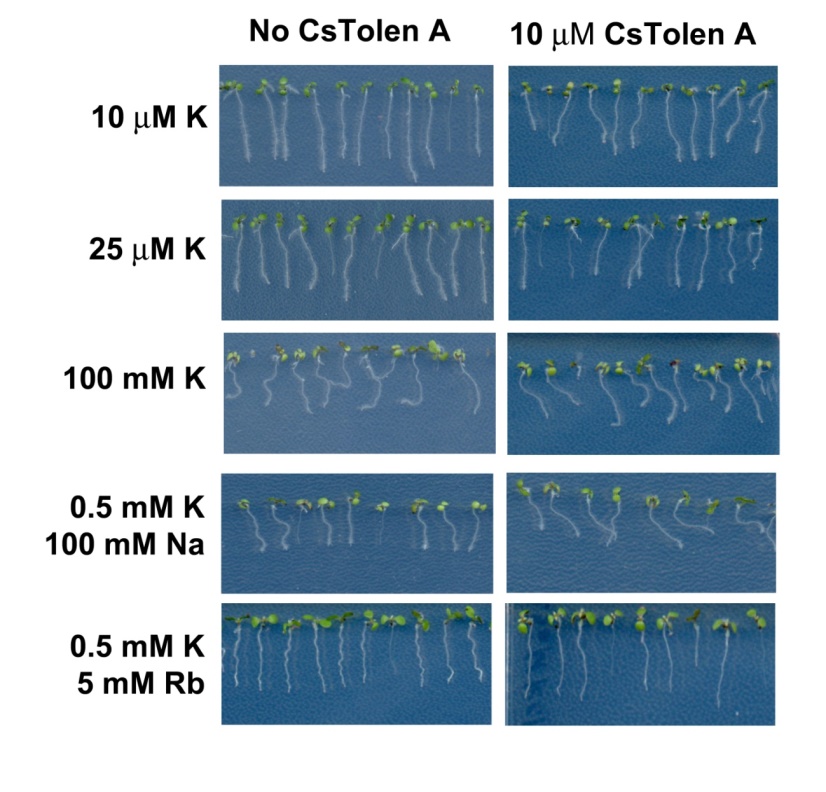


**Supplementary Figure 6. Images of Col-0 treated with nutrient stress and CsTolen A.** Plants were germinated and grown on K^+^ deficiency (10 and 25 μM), excess K^+^ (100 mM) and suboptimal (0.5 mM) KCl media in the presence of 100 mM NaCl or 5 mM RbCl and 10 μM of CsTolen A for 8 days.


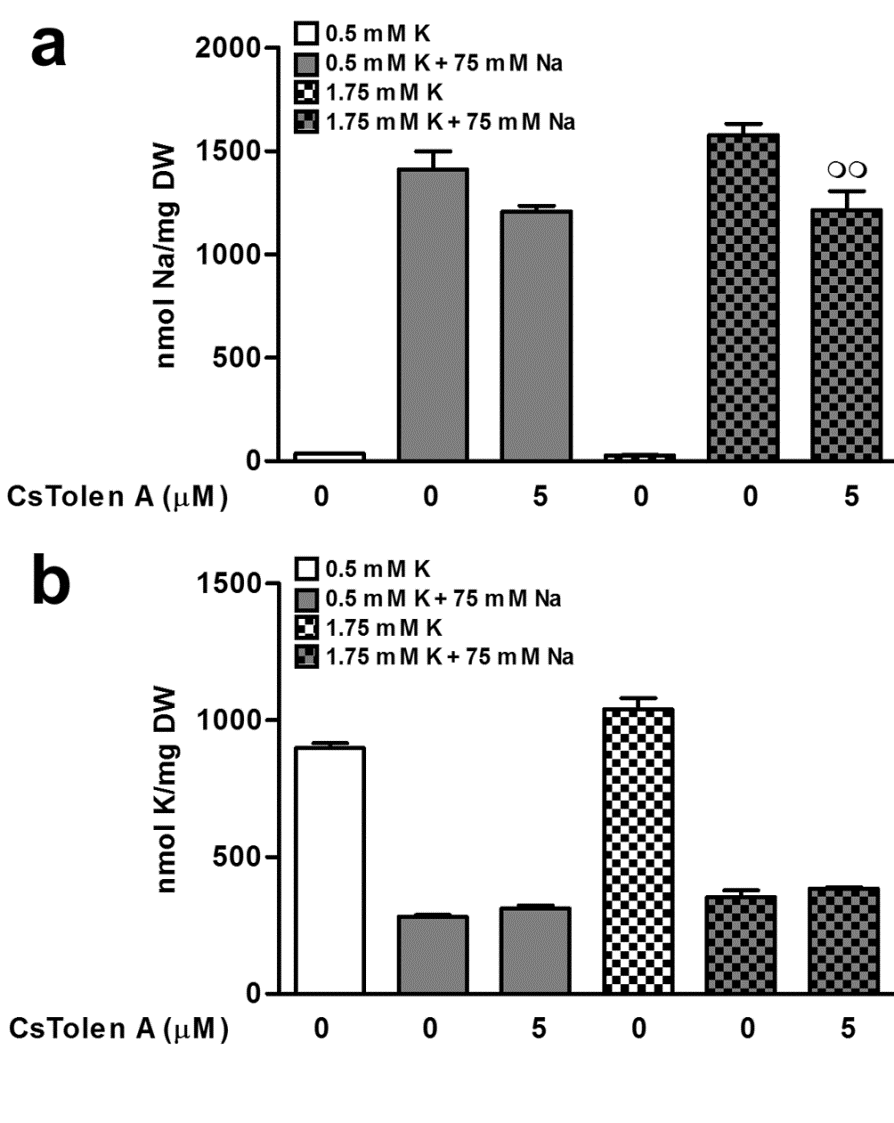


**Supplementary Figure 7. Na^+^ and K^+^ concentrations in Col-0 treated with CsTolen A.** (**a**) Na^+^ and (**b**) K^+^ concentrations in plants grown in salt stress. Plants germinated and grown on suboptimal (0.5 mM) or optimal (1.75 mM) KCl media in the presence or absence of 75 mM NaCl and 5 μM of CsTolen A for 8 days were dried and extracted. White circles indicate statistically significant difference compared to the non-CsTolen control: 1.75 mM KCl + 75 mM NaCl (n=3, *P* < 0.01).


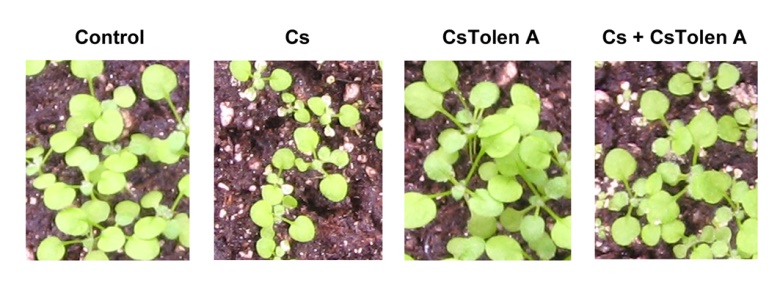


**Supplementary Figure 8. Images of soil-grown 15-day-old Col-0 plants treated with Cs^+^ and CsTolen A.** Plants were germinated and grown on soil containing 12.96 mmol CsCl and 0.92 μmol CsTolen A kg^-1^ dry soil. Seven-day-old seedlings were sprayed with 10 μM CsTolen A.
